# Gradual Reduction Using Overhead Traction for Late-Detected Developmental Dysplasia of the Hip: A Report of Three Cases Diagnosed Among Children Over Four Years Old

**DOI:** 10.7759/cureus.63833

**Published:** 2024-07-04

**Authors:** Kenichi Mishima, Yasunari Kamiya, Kenta Sawamura, Masaki Matsushita, Shiro Imagama

**Affiliations:** 1 Department of Orthopaedic Surgery, Nagoya University Graduate School of Medicine, Nagoya, JPN

**Keywords:** developmental dysplasia of the hip (ddh), pediatric hip, oht, avascular necrosis (avn), gradual reduction, overhead traction, late-detected

## Abstract

Optimal reduction methods for late-detected developmental dysplasia of the hip (DDH) remain debatable. Gradual reduction (GR) using traction is a safer and more reliable option for late-detected DDH than closed reduction or open reduction with or without preliminary traction. GR using overhead traction, one of the current GR methods, has been indicated for children of walking age up to four years of age, whereas the upper age limit of this method has not yet been determined. We present three cases of late-detected DDH whose hips were treated between four and six years of age with this method. Stable reduction without subsequent redislocation was technically accomplished for all patients, albeit the duration of horizontal traction became longer than usual. Clinically significant avascular necrosis (AVN) has developed in children aged ≥5 years, indicating the need for some modifications to the conventional protocol to prevent AVN.

## Introduction

Initial management of developmental dysplasia of the hip (DDH) involves correction of malposition of the femoral head relative to the acetabulum [1]. Long-term survival of the involved hips entails anatomical preservation of the proximal femoral geometry [2]. Accordingly, successful treatment of DDH has been defined as the accomplishment of a congruent and concentric hip joint without avascular necrosis (AVN) at skeletal maturity [3]. The use of the Pavlik harness has been commonly indicated for children under six months of age [4]. This method has produced satisfactory outcomes with significantly lower rates of AVN and satisfactory reduction rates when applied appropriately [5]. For cases of failed Pavlik harness treatment or children aged six months or older, closed reduction (CR) or open reduction (OR) with or without preliminary traction has been implemented for DDH [6]. Far from CR and OR, gradual reduction (GR) using traction seems to have occupied the third position in the treatment of DDH, presumably because of the economic, social, and emotional burdens of prolonged traction. Since 1976, we have utilized current GR using overhead traction (OHT) for cases of failed Pavlik harness treatment or children aged six months or older, and have already reported satisfactory long-term outcomes with a low incidence of AVN [7]. In contrast, the upper age limit for this method has not yet been determined because we have hitherto applied it for children under four years of age at presentation. In this paper, we report the cases of three children with DDH whose hips were treated between four and six years of age with GR using OHT.

## Case presentation

Gradual reduction using overhead traction

**Figure 1:**
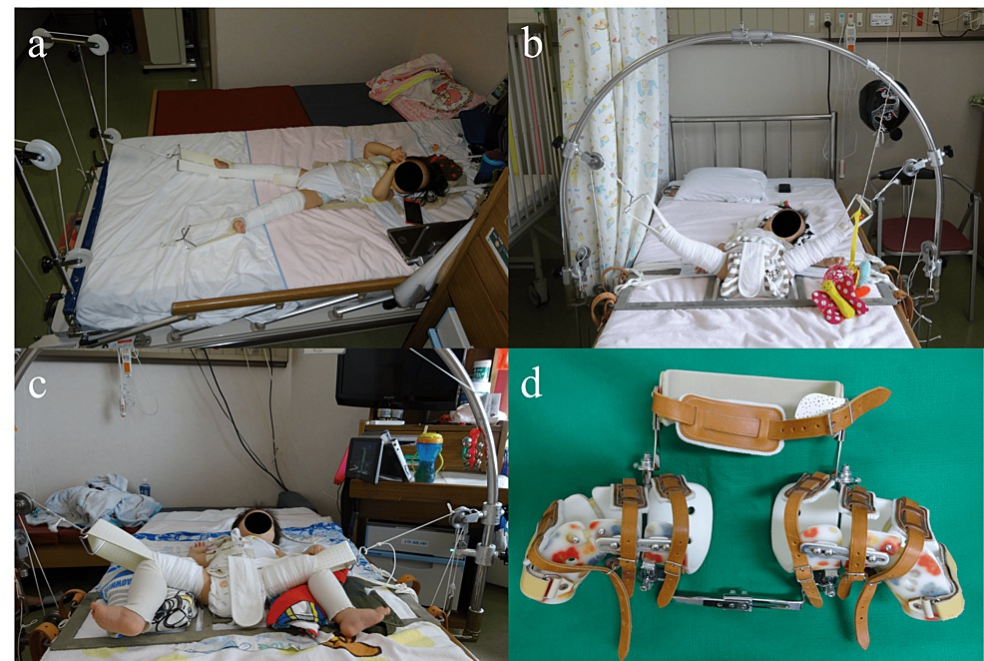
Photographs illustrating each step of gradual reduction using overhead traction. (a) Horizontal skin traction. (b) Vertical traction (overhead traction). (c) Above-knee traction. (d) Variable custom-made brace.

The current procedure comprises the following three steps: horizontal skin traction in a slightly abducted position (Figure 1a), vertical traction (overhead traction) with the knees extended (Figure 1b), and above-knee traction (Figure 1c), as described previously [7]. The first step is to stretch the muscles and soft tissues surrounding the hip joint and achieve an acceptable descent of the femoral head. Unless the center of the capital femoral physis is leveled with or below Hilgenreiner’s line on radiographs following the initial four-week traction, which corresponds to Yamamuro’s “distance a” of ≥0 mm [8], traction is continued in hospitals with heavier weights. In the second step, we employ a dedicated apparatus to gradually abduct the hips while maintaining them at 100° of flexion. In the third step, the knees are allowed to flex actively to reduce the muscle tone of the hip adductors and hamstrings. After arthrography is performed under general anesthesia, the reduced hip is immobilized in the most concentric position using a double hip spica cast for five weeks, followed by the application of a variable custom-made brace allowing for alterations in ﬂexion-abduction-rotation configuration of the hips (Figure 1d) for 24 hours for three months to achieve concentric reduction [9]. Specifically, the hips are stabilized in the human or Lorenz position during the first month. In the second month, the brace is fitted in the Lange position with the hips flexed less than 90° and internally rotated to some extent. Finally, the backrest is detached to allow children to roll and crawl freely.

Case 1

**Figure 2:**
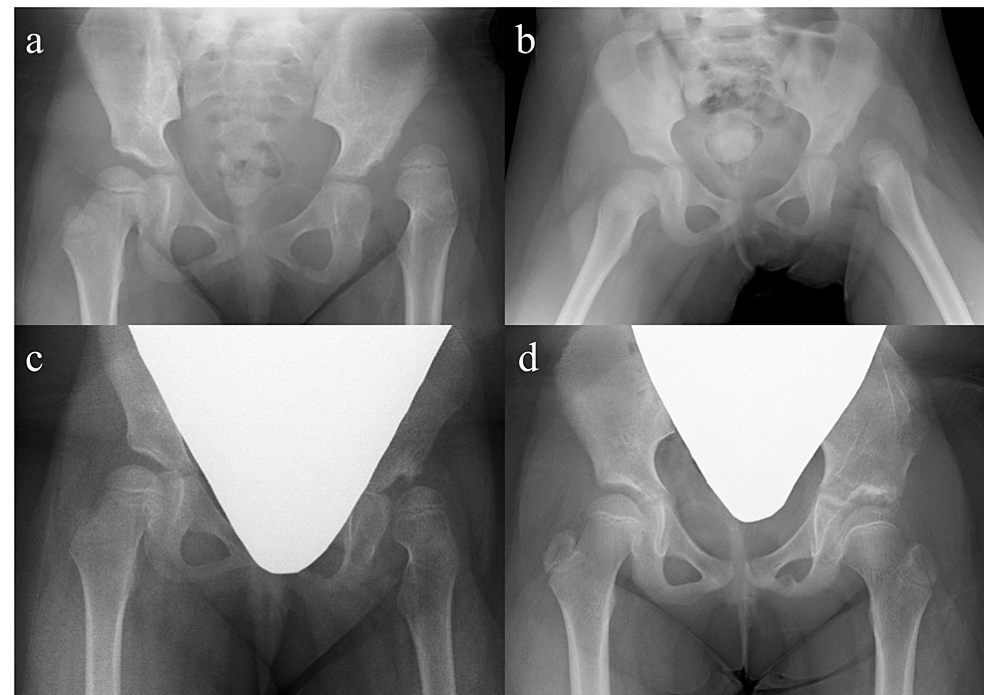
Supine anteroposterior hip radiographs of Case 1. (a) An initial hip radiograph obtained at four years and eight months of age showing a left-sided developmental dysplasia of the hip with Yamamuro’s “distance a” of −5 mm. (b) A follow-up hip radiograph obtained after 37 days of horizontal skin traction showing an acceptable “distance a” of 1 mm. (c) A follow-up hip radiograph obtained after treatment with a flexion-abduction brace showing the residual acetabular dysplasia of the left hip. (d) The latest hip radiograph obtained four years after Salter innominate osteotomy demonstrating a mild coxa magna with a spherical and congruent femoral head of the left hip.

A girl aged four years and eight months girl presented with a three-day history of left hip pain. Anteroposterior hip radiographs revealed a left hip dislocation and a dysplastic left acetabulum with Yamamuro’s “distance a” of −5 mm (Figure 2a). She underwent horizontal skin traction for 37 days using up to 4.5 kg of weight for each leg, leading to an acceptable “distance a” of 1 mm (Figure 2b). After seven days of vertical traction, spontaneous reduction of the hip occurred immediately after the transition to 14 days of above-knee traction. Salter innominate osteotomy (SIO) was performed to correct the residual acetabular dysplasia at five years and four months of age (Figure 2c). Four years after SIO, the development of a mild coxa magna with a spherical and congruent femoral head was observed on radiographs (Figure 2d).

Case 2

**Figure 3:**
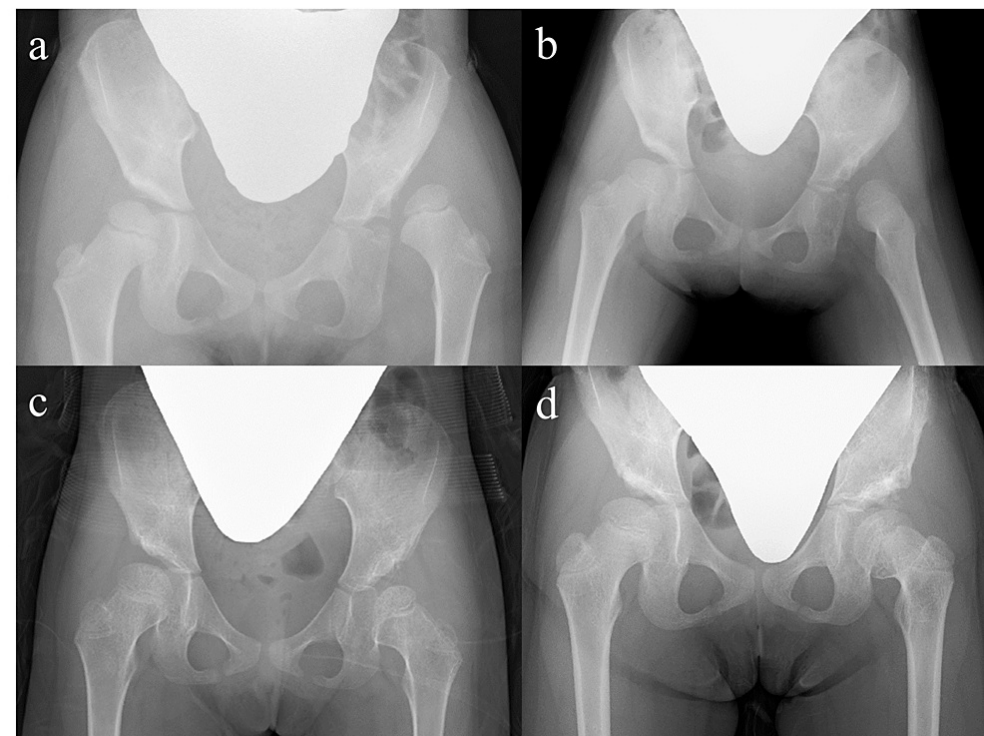
Supine anteroposterior hip radiographs of Case 2. (a) An initial hip radiograph obtained at five years and zero months of age showing a left-sided developmental dysplasia of the hip with Yamamuro’s “distance a” of −7 mm. (b) A follow-up hip radiograph obtained after 49 days of horizontal skin traction showing a sufficient “distance a” of 0 mm. (c) A follow-up hip radiograph obtained after treatment with a flexion-abduction brace showing the residual acetabular dysplasia of the left hip. (d) The latest hip radiograph obtained three years after Salter innominate osteotomy (SIO) demonstrating an apparent coxa vara with shortening of the femoral neck. The femoral head is slightly enlarged but seems to preserve sphericity. She underwent SIO for the acetabular dysplasia of the right hip at eight years and four months of age.

A five-year-old girl presented for evaluation of gait abnormalities. Radiographs of the hip showed a severely dislocated femoral head with a steep acetabulum of her left hip, showing Yamamuro’s “distance a” of −7 mm (Figure 3a). She underwent horizontal skin traction for 49 days using up to 3.5 kg of weight for each leg, resulting in a sufficient “distance a” of 0 mm (Figure 3b). After five days of vertical traction, spontaneous reduction of the hip occurred immediately after the transition to 12 days of above-knee traction. SIO was performed to correct the residual acetabular dysplasia at five years and 10 months of age (Figure 3c). Three years after SIO, radiographs demonstrated apparent coxa vara with femoral neck shortening. The femoral head was slightly enlarged but seemed to preserve sphericity (Figure 3d).

Case 3

**Figure 4:**
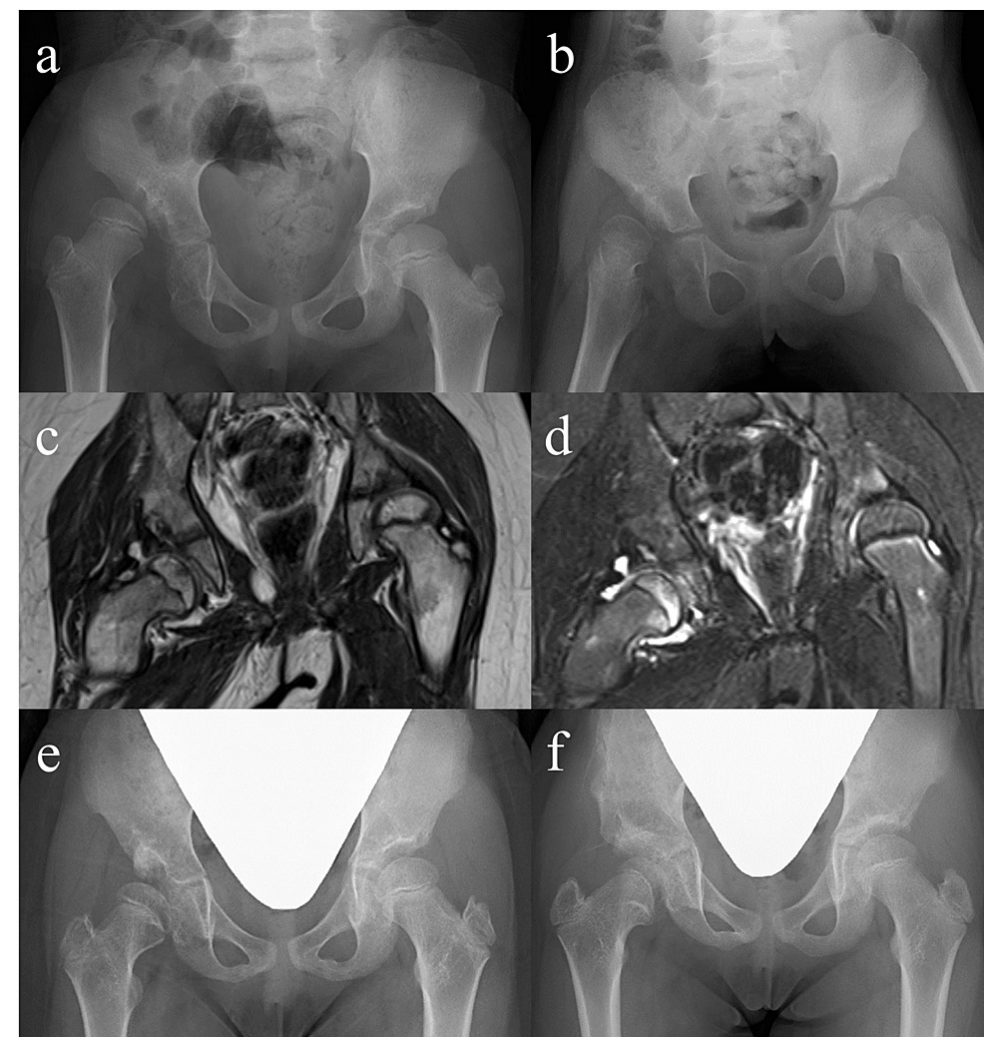
Supine anteroposterior hip radiographs and MRI images of Case 3. (a) An initial hip radiograph obtained at five years and 11 months of age showing a right-sided developmental dysplasia of the hip with Yamamuro’s “distance a” of −10 mm. (b) A follow-up hip radiograph obtained after 48 days of horizontal skin traction showing a decent “distance a” of −1 mm. (c and d) T2-weighted coronal (c) and short-tau inversion recovery coronal (d) MRI images obtained at the initiation of treatment with a ﬂexion-abduction brace demonstrating intracapsular effusions and edematous changes involving the medial portion of the capital femoral epiphysis and metaphysis. (e) A follow-up hip radiograph obtained after treatment with a ﬂexion-abduction brace showing the residual acetabular dysplasia of the right hip. (f) The latest hip radiograph obtained two and a half years after Salter innominate osteotomy demonstrating an obvious coxa vara with a shortened femoral neck and a slightly aspherical femoral head. Note a thin osteosclerotic line at the medial aspect of the femoral neck, possibly representing transient growth arrest after reduction.

A girl aged five years and 11 months presented for assessment of gait disturbance. Radiographs showed a high hip dislocation with a vertically inclined acetabular roof of the right hip, with Yamamuro’s “distance a” approaching −10 mm (Figure 4a). She underwent horizontal skin traction for 48 days using up to 4.5 kg of weight for each leg, reaching a decent “distance a” of −1 mm (Figure 4b). After four days of vertical traction, spontaneous reduction of the hip occurred immediately after the transition to 19 days of above-knee traction. MRI performed at the initiation of bracing showed intracapsular effusions and edematous changes involving the medial portion of the capital femoral epiphysis and metaphysis (Figures 4c, 4d). SIO was performed to correct the residual acetabular dysplasia at seven years and one month of age (Figure 4e). Two and a half years after SIO, obvious coxa vara with a shortened femoral neck and a slightly aspherical femoral head were observed on radiographs. In addition, a thin osteosclerotic line was visualized at the medial aspect of the femoral neck, possibly representing a transient growth arrest after reduction (Figure 4f).

## Discussion

**Figure 5:**
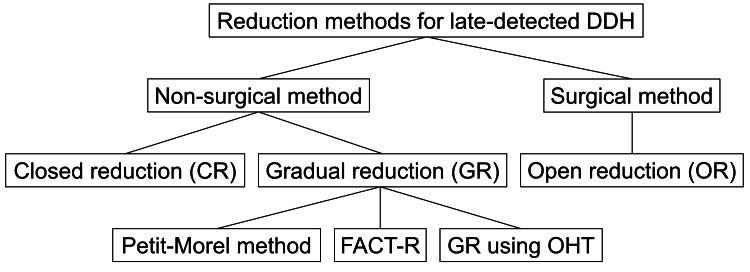
A classification of reduction methods available for late-detected developmental dysplasia of the hip.

Currently, the available GR is classified into three types based on the combination of traction modes (Figure 5). The Petit-Morel (PM) method, a pioneering method of GR, involves only horizontal longitudinal traction with both knees extended, having already demonstrated a lower incidence of AVN than CR and OR [10]. FACT-R, an ultrasound-guided gradual reduction using continuous flexion and abduction traction, employs horizontal transverse (flexion and abduction) traction in addition to horizontal longitudinal traction. GR using OHT consists of three modes of traction, namely, horizontal longitudinal, overhead (vertical), and above-knee (horizontal transverse) traction [7]. We have long prioritized the avoidance of AVN over shortening the treatment period in the management strategy for DDH because residual acetabular dysplasia or hip subluxation can be addressed using acetabular redirectional osteotomies or acetabuloplasty with or without femoral realignment osteotomy. In contrast, surgical restoration of the sphericity of the femoral head, which is distorted and enlarged as a sequela of AVN, remains challenging because of the irreversibility of cartilaginous tissue damage. Since 1976, we have utilized the current GR using OHT for cases of failed Pavlik harness treatment or children aged six months or older. The oldest patient to whom this method was performed was a girl who initially presented at four years and zero months of age [7]. As for the other GR methods using traction, the FACT-R method has been applied only to infants younger than one year old in the English literature [11]. A previous investigation using the PM method included a patient aged 4.9 years at the onset of treatment; however, it is unclear whether closed reduction with traction succeeded without AVN [10]. Here, we report three patients aged four to six years at diagnosis who underwent GR using the OHT method. Stable closed reduction was achieved for a severely dislocated hip with International Hip Dysplasia Institute (IHDI) grade IV [12] by extending the period of horizontal traction beyond the usual duration of four weeks.

In our previous study, the incidence of AVN following GR using OHT was only 2.7%, which roughly coincided with those following the FACT-R (1.0%) and PM (2.1%) [7,10,11]. In this case series, morphological features of AVN were observed in all three cases during follow-up. Case 1 had secondary coxa magna without appreciable physeal damage. The sphericity of the ossific nucleus was maintained and rated as grade I according to the Kalamchi and MacEwen and Bucholz and Ogden classifications [13,14]. Cases 2 and 3 had developmental coxa vara and femoral neck shortening caused by medial physeal damage, with or without loss of sphericity of the epiphysis. These were rated as Bucholz and Ogden grade IV, similar to one AVN hip in our previous cohort [7]. Interestingly, both patients complained of stretch pain in the adductor longus during vertical traction (overhead traction). After reduction, they exhibited swelling and livedo reticularis-like skin discoloration from the inguinal region to the proximal thigh for several days, suggesting regional congestion associated with acute arthritis of the hip and surrounding soft tissue inflammation caused by a rapid and intense reduction. In the FACT-R method, AVN occurred in one infant following abrupt spontaneous reduction during above-knee traction, whose traction period was shorter than usual [11]. Considering possible precautions against AVN, such as prior adductor tenotomy, the addition of Bryant’s vertical traction in the second step before abducting the hips [15], a gradual increase in knee flexion during the third step, and a slower incremental decrease in weight after reduction, may be necessary, especially for children aged five years, to suppress an impact of reduction on pathological intracapsular structures, including the hypertrophic pulvinar, ligamentum teres, and limbus, and a sustained inflammatory reaction induced inside and outside the reduced hip.

## Conclusions

Although the duration of horizontal traction was longer than usual, stable CR was technically feasible, even in patients aged ≥4 years with the original GR using the OHT method. However, clinically significant AVN developed in patients aged ≥5 years, indicating the need for modifications to the conventional protocol for successful treatment.
